# The complete chloroplast genome sequence of *Abies recurvata* Mast. from southwest China: insights into genome features and evolutionary relationships

**DOI:** 10.1080/23802359.2026.2643992

**Published:** 2026-05-11

**Authors:** Yi-Zhen Shao, Xiao-Hang Li, Meng-Yao Zhang, Sheng-Qian Guo, Yun Chen, Zhi-Liang Yuan, Shi-Chang Zhang, Peng-Fei Zhao

**Affiliations:** College of Life Sciences, Henan Agriculture University, Zhengzhou, China

**Keywords:** *Abies chensiensis*, *Abies delavayi*, genomic comparisons, phylogeny reconstruction, whole plastid genome

## Abstract

*Abies recurvata* Mast. is an endemic and vulnerable conifer species in China. To date, its taxonomic position relative to related taxa (e.g. *A. delavayi* and *A. chensiensis*) remains ambiguous. To our knowledge, this work presents the first full chloroplast genome assembly and comprehensive characterization of *Abies recurvata* (Purple-coned Fir). This genome exists as a circular molecule of 120,063 bp, harboring 113 distinct genes. Additionally, 66 microsatellite repeats and 12 tandem repeats were identified. Phylogenetic analysis challenged conventional taxonomic views, revealing that *A. recurvata* is distantly related to *A. delavayi* and *A. chensiensis*, but instead forms a strongly supported clade with *A. fargesii*.

## Introduction

*Abies recurvata* Mast. is an endemic fir species in China, occurring in Tibet, southern Gansu, and northern to northwestern Sichuan at elevations of 2300–3600 m (Kuan 1981; Farjon 1990). *A. recurvata* yields timber noted for exceptional solidity and durability, offering superior quality relative to many firs (Liepelt et al. 2010; Liu et al. 2025). This makes it applicable to construction, furniture manufacturing, and wood-fiber industrial uses (Farjon and Rushforth 1989; Xi et al. 2021). *Abies recurvata* is categorized as vulnerable (VU) on the IUCN Red List of Threatened Species, yet no comprehensive characterization of its complete chloroplast genome has been conducted to date.

The taxonomic status of *Abies recurvata* has long posed challenges in classical taxonomy, especially concerning its relationships with *A. chensiensis* Tiegh., *A. delavayi* Diels, *A. delavayi* subsp. *fansipanensis* (Q.P.Xiang, L.K.Fu & Nan Li) Rushforth and *A. fabri* (Mast.) Craib (Dallimore and Jackson 1966; Shao et al. 2020). Historically, for instance, *A. chensiensis* have repeatedly been treated as varieties or subspecies of *A. recurvata* (Handel-Mazzetti 1929; Chen et al. 2017). Although several chloroplast genome-based phylogenetic studies of *Abies* have been published, most have focused on broader genus-level frameworks and have not fully incorporated all species closely related to *A. recurvata* (Fu et al. 2019; Guo, Yang, Bai, et al. 2021; Guo, Yang, Li, et al. 2021; Shao et al. 2023). Recent breakthroughs in whole-genome sequencing technologies have propelled the application of plastid genomes toward the accurate reconstruction of phylogenetic evolutionary relationships (Guo et al. 2019; He et al. 2019; Ran et al. 2020; Song et al. 2024; Cao et al. 2025). Plastid genomes feature conserved structure, no recombination, and abundant informative sites, making them reliable for resolving long-standing taxonomic uncertainties (Miao et al. 2017; Fu et al. 2019; Li et al. 2023; Lai et al. 2024; Xie et al. 2025).

In this study, we performed sequencing, assembly, and systematic characterization of the complete chloroplast genome of *A. recurvata*, alongside comparative genomic analyses involving phylogenetically related species.

## Materials and methods

### Plant material collection and genomic DNA extraction

**Figure 1.**
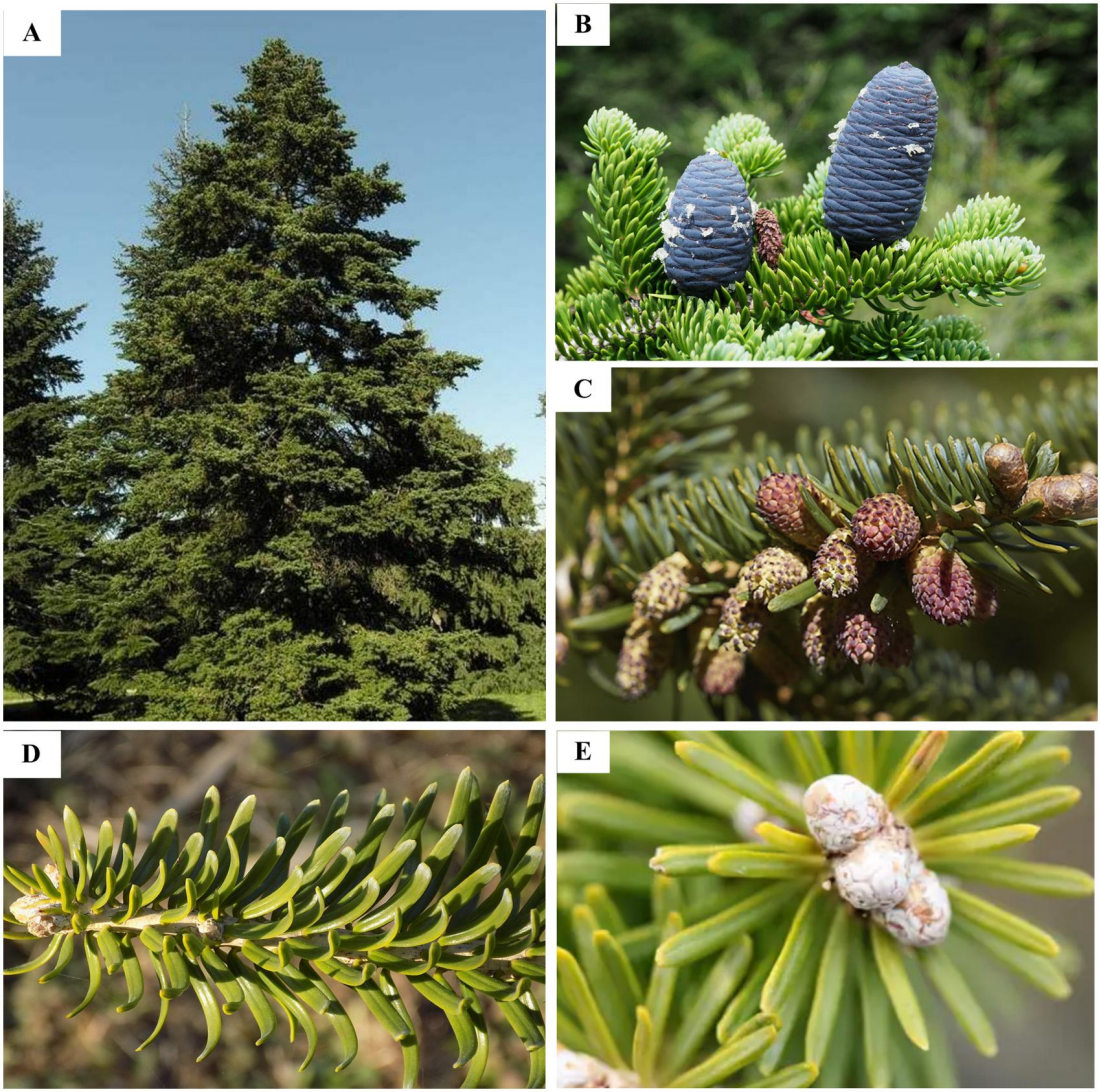
Morphological characteristics of *Abies recurvata*. (A) Whole plant. (B) Mature seed cones. (C) Pollen cones. (D) Leaves. (E) Branch. All photographs were taken by Yi-Zhen Shao and Qiao-Ping Xiang. Main characteristics: bark irregularly plated; young shoots yellowish-tomentose; cones purplish, ellipsoid- to cylindrical-ovoid, with median seed scales reniform-flabellate and bract scales exserted.

Leaf samples of *Abies recurvata* were gathered by Xian-Chun Zhang in Daofu County, Sichuan Province, China (30.98°N, 101.13°E) (Figure 1). A voucher specimen (No. 5828) has been permanently archived in the herbarium maintained by the Institute of Botany, Chinese Academy of Sciences (abbreviation: PE; correspondence: Qiao-Ping Xiang, qpxiang@ibcas.ac.cn) (Figure S1).

### Genome sequencing, assembly, and annotation

Paired-end libraries were prepared with the TruSeq Nano kit and sequenced on HiSeq X (2 × 150 bp) (Yan et al. 2020). Reads were trimmed and Velvet-assembled (*Q* ≥ 5, *N* ≤ 10%) (Zerbino and Birney 2008; Xue et al. 2024). The generated chloroplast genome was subjected to comprehensive annotation using the GeSeq tool and tRNAscan-SE v1.3.1 program (Schattner et al. 2005; Tillich et al. 2017), while supplementary validation work was carried out with the CLC Assembly Cell software (CLC Bio, Aarhus, Denmark). Critical gene structural elements, such as the locations of start/stop codons and the junctions between exons and introns, were manually checked in Geneious and Sequin by referencing published reference plastome sequences (Kearse et al. 2012; Lohse et al. 2013). The annotated plastome sequence has been submitted to the GenBank database and assigned the accession number MH706712.

### Repeat sequences detection and comparative genomic analysis

Simple sequence repeats (SSRs) were detected by means of the MISA bioinformatics pipeline, with threshold criteria defined according to motif length: with the screening criteria set as follows: a microsatellite locus was considered valid if it contained no fewer than 10 repeat units for a mononucleotide motif, at least 5 for a dinucleotide motif, a minimum of 4 for a trinucleotide motif, and no less than 3 for tetra-, penta-, or hexanucleotide motifs. Long repetitive elements were further examined with REPuter (Kurtz et al. 2001), with a minimum repeat size of 30 bp, a maximum length of 50 bp, and a Hamming distance tolerance of up to three mismatches permitted. For comparative plastome analyses, we retrieved the complete chloroplast genomes of *A. chensiensis* (MH706706), *A. delavayi* (MH706709), *A. delavayi* subsp. *fansipanensis* (MH706720), *A. fabri* (MH706710), and *A. fargesii* (MH706716) from the NCBI database. Subsequently, multiple genome alignment and visual analysis were performed via the mVISTA software, adopting the Shuffle-LAGAN alignment algorithm. (Frazer et al. 2004).

### Phylogenetic reconstruction

The phylogenetic tree was reconstructed using the complete chloroplast genome data from 23 accessions, which encompassed 19 fir species, spanned major clades focused on the genus *Abies* and encompassed all taxa that are phylogenetically proximate to *A. recurvata* (Xiang et al. 2018). *Keteleeria davidiana* (Bertr.) Beissn. was chosen as the outgroup. Alignment of multiple genome sequences was accomplished. Sequence alignment was implemented in MAFFT v.7 (Katoh and Standley 2013). Phylogenetic inference was then executed under a maximum-likelihood (ML) framework in RAxML v.8.1, utilizing the GTR + GAMMA model for nucleotide substitutions. Branch support values were determined through 1000 bootstrap iterations. Detailed information for chloroplast genome sequences analyzed herein is provided in Supplementary Table S1.

## Results

### Chloroplast genomic characteristics of *A. recurvata*

**Figure 2.**
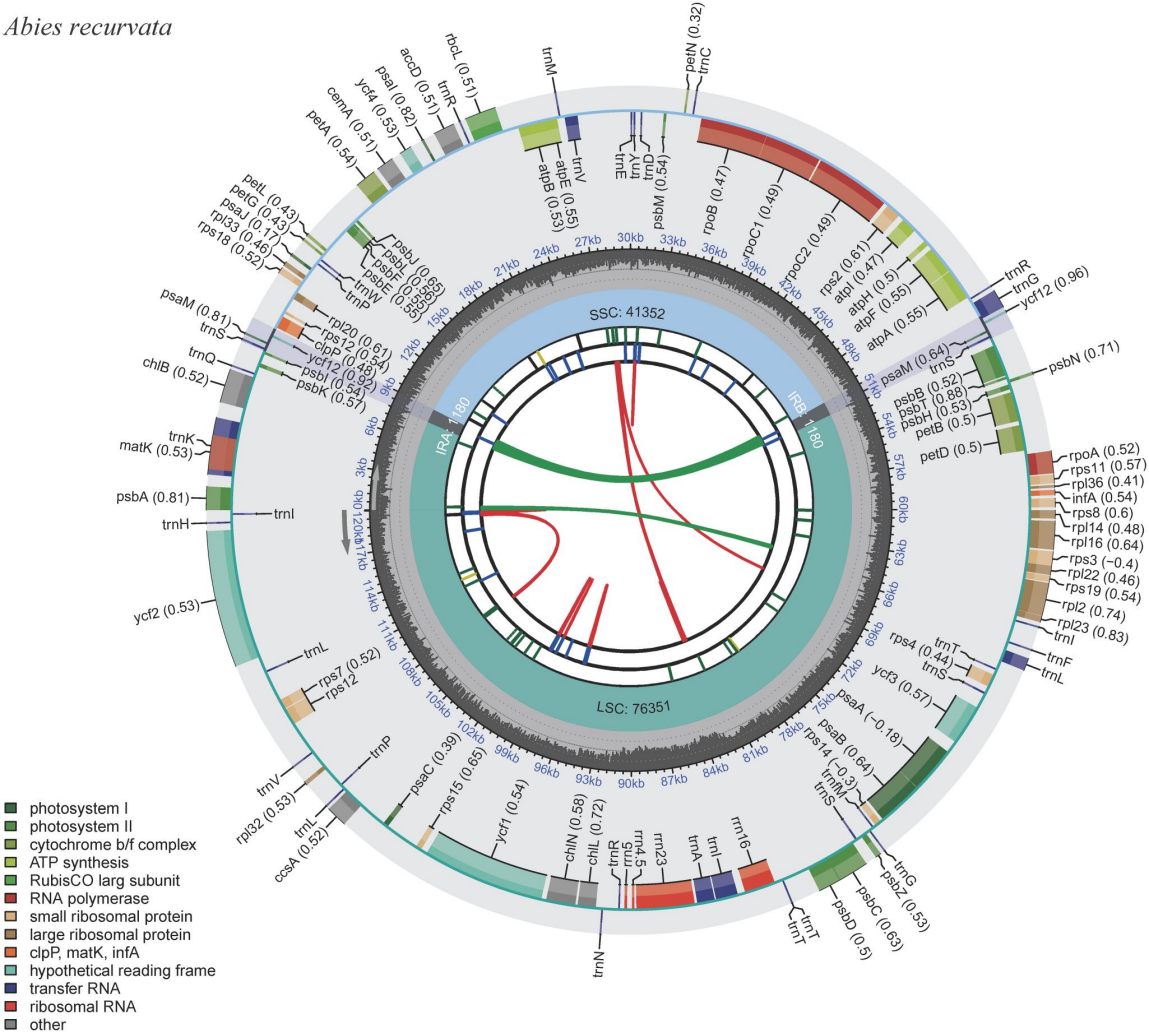
Schematic representation of the chloroplast genome of *Abies recurvata*. Genes are color-coded according to their functional groups. Genes drawn outside the circle are transcribed clockwise, while those inside are transcribed counter-clockwise. The inner gray tracks depict nucleotide composition, with darker shades indicating GC content and lighter shades representing AT content.

The complete cp genome of *A. recurvata* was 120,063 bp in length, with mean sequencing depths of 280× (Figure S2), and exhibited the canonical quadripartite organization: a large single-copy (LSC) region spanning 65,351 bp, a small single-copy (SSC) region of 54,184 bp, and two identical inverted repeat (IR) regions (each spanning 264 bp). The chloroplast genome displays an overall GC content of 38.30%. Annotation work led to the identification of 113 distinct genes (Table S2 and Figure 2). Each IR region is defined by the presence of two specific tRNA genes, namely *trn*I-CAU and *trn*T-GGU. Analysis of intron–exon structures revealed that 11 protein-coding genes (e.g. *atp*F, *rps*12, *rpl*16, *pet*B, *pet*D, and *rpo*C1) each contain one intron, while two genes (*clp*P and *ycf*3) contain two introns (Figure S3). Additionally, a palindromic IR sequence of approximately 1180 bp, spanning the ‘*ycf*12-*trn*S-*psa*M-*trn*G’ region, was identified near the LSC/IR boundary.

### Repeat sequence analysis

A total of 66 SSRs were screened from the chloroplast genome of *A. recurvata*, among which mononucleotide SSRs constituted the dominant type, accounting for 42 in total. They were followed by dinucleotide repeats (12), tetranucleotide repeats (7), trinucleotide repeats (2), and pentanucleotide repeats (1), which represented the least abundant category (Figure S4). Mononucleotide repeats accounted for the majority (63.60%), whereas pentanucleotide repeats represented the smallest proportion (1.50%). The trinucleotide SSRs consisted of two motif types (ATT and AAT), while the tetranucleotide repeats comprised 12 types. Two pentanucleotide motifs were also detected: AATCG, and ATTCG (Figure S4). Furthermore, 47 putative long repetitive sequences were identified in total within the plastome, falling into three distinct categories: 21 forward repeats, 14 palindromic repeats, and 12 tandem repeats (Figure S4).

### Comparative chloroplast genomics

Comparative analysis of six chloroplast genomes yielded two key observations: first, the IR regions possess high sequence conservation; second, this conservation level far exceeds. Sequence divergence is predominantly localized within the non-coding regions of the LSC and SSC. Virtually all sequence variations are primarily across intergenic spacers and other non-coding genomic intervals. Whole-genome alignment revealed that among all annotated genes, only *ycf*12 exhibited substantial sequence divergence (Figure S5).

### Phylogenetic relationships and implications

**Figure 3.**
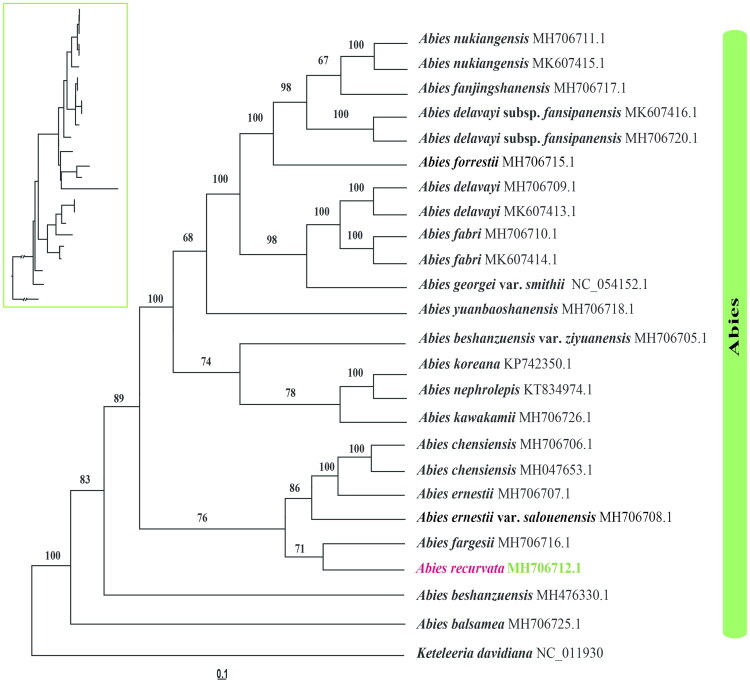
Maximum-likelihood (ML) phylogram based on 23 complete chloroplast genomes from 19 *Abies* species, with *Keteleeria davidiana* as the outgroup. Bootstrap support values are labeled at each node. Species names and GenBank accession numbers are given at the branch tips. The target species of this study, *A. recurvata*, is highlighted in bold and in color. A phylogram-style tree is shown in the upper-left corner. The following sequences were used: *A. balsamea* MH706725 (Wu et al. 2019), *A. beshanzuensis* MH476330 (Shao et al. 2018), *A. beshanzuensis* var. *ziyuanensis* MH706705 (Fu et al. 2019), *A. chensiensis* MH706706 and MH047653 (Liu et al. 2018; Su et al. 2019), *A. delavayi* MH706709 and MK607413 (Shao et al. 2020), *A. delavayi* subsp. *fansipanensis* MH706720 and MK607416 (Shao et al. 2020), *A. ernestii* MH706707 (Shao et al. 2022), *Abies ernestii* var. *salouenensis* MH706708 (Shao et al. 2023), *A. fabri* MH706710 and MK607414 (Shao et al. 2020), *A. fargesii* MH706716 (Guo et al. 2019), *A. fanjingshanensis* (Guo et al. 2019), *A. forrestii* MH706715 (Dong et al. 2021), *A. georgei* var. *smithii* NC_054152 (Li et al. 2020), *A. kawakamii* MH706726 (Shao et al. 2019), *A. koreana* KP742350 (Yi et al. 2015), *A. nephrolepis* KT834974 (Yi et al. 2016), *A. nukiangensis* MH706711 and MK607415 (Shao et al. 2020), *A. yuanbaoshanensis* MH706718 (Zhang et al. 2020), and *Keteleeria davidiana* NC_011930 (www.ncbi.nlm.nih.gov/).

Phylogenetic tree reconstruction conducted on the basis of complete chloroplast genome sequences from 23 taxa—with *Keteleeria davidiana* selected as the outgroup—uncovered that all representative species belonging to the genus *Abies* formed a monophyletic clade with strong statistical support (BSML = 100) (Figure 3). Within this monophyletic clade, the North American endemic *A. balsamea* and East Asian congeners of *Abies* formed a well-defined sister clade (BSML = 83). In the East Asian subclade, *A. recurvata* clustered with *A. fargesii* (BSML = 71); this subclade further grouped with *A. chensiensis* (BSML = 76), but exhibited a relatively distant phylogenetic affinity to *A. delavayi*.

## Discussion and conclusions

The chloroplast genome of *A. recurvata* exhibits typical Pinaceae features, including a quadripartite structure, conserved GC content, and the absence of functional ndh genes—a trait indicative of possible functional substitution within the family (Blazier et al. 2011; Yuan et al. 2024). A palindromic repeat identified near the 52‑kb inversion site is consistent with previous reports in related species (Shao et al. 2022), while the prevalence of mononucleotide SSRs implies their contribution to intraspecific genetic diversity (Kaur et al. 2015). Comparative genomic analyses further underscore the strong conservation of IR regions, with sequence divergence predominantly accumulating in non‑coding regions. This structural pattern may elucidate the historical challenge in developing diagnostic molecular markers for closely related *Abies* taxa (Yin et al. 2017; Xiang et al. 2018; Zhao et al. 2023). Notably, among examined loci, *ycf*12 displayed marked sequence divergence, highlighting its promise as a novel chloroplast marker, whereas several conventional loci (e.g. *mat*K, *rpl*16, *rps*18, *trn*C‑D, and *trn*S‑G) exhibited limited phylogenetic resolution (Zhou et al. 2022).

With the rapid development of genomic technologies, whole-genome data have been far more extensively applied in phylogenetic and evolutionary research endeavors (Yuan et al. 2018; Zhang et al. 2019). Our analysis conclusively resolves the long‑debated systematic position of *A. recurvata*, robustly placing it within a clade containing *A. fargesii*, and distinctly separate from *A. chensiensis*, *A. delavayi*, *A. delavayi* subsp. *fansipanensis*, and *A. fabri*. This finding affirms the utility of complete chloroplast genomes in clarifying complex phylogenetic relationships within *Abies* (Yue et al. 2023; Zhang et al. 2025).

## Supplementary Material

Supplementary material.doc

## Data Availability

The reported genome has been deposited in the NCBI GenBank database (No. MH706712). The associated BioProject, SRA, and BioSample identifiers are PRJNA790666, SRP351768, and SAMN24219953, respectively.
