# Supplementary material for: The complete chloroplast genome sequence of Abies recurvata Mast. from southwest China: insights into genome features and evolutionary relationships
Source: Mitochondrial DNA B Resour. 2026 May 11;11(6):727–33. doi: 10.1080/23802359.2026.2643992 (PMC13162563; doi:10.1080/23802359.2026.2643992)
Supplement: Supplementary material.doc [file TMDN_A_2643992_SM7411.doc]

**Supplementary material**

The following supplementary tables and figures are available online:

**Table S1.** List of complete chloroplast genomes analyzed in this study.

**Table S2.** Gene organization of the *Abies recurvata* chloroplast genome.

**Figure S1.** The illustration of *Abies recurvata* specimen preserved at the herbarium of the Institute of Botany, Chinese Academy of Sciences (PE). Its key taxonomic characteristics are as follows: bark irregularly plated; young shoots yellowish-tomentose; cones purplish, ellipsoid- to cylindrical-ovoid, with median seed scales reniform-flabellate and bract scales exserted.

**Figure S2**. Coverage depth of the *Abies recurvata* chloroplast genome assembly, with an average of 280.58×, a maximum of 611×, and a minimum of 47×.

**Figure S3.** Schematic representation of cis- (A) and trans-splicing (B) genes in the chloroplast genome of *A. recurvata*.

**Figure S4.** Distribution and abundance of simple sequence repeats (SSRs; A, B) and long repeats (C, D) in the *A. recurvata* chloroplast genome.

**Figure S5.** Whole‑genome alignment of six chloroplast genomes performed with mVISTA, using *A. chensiensis* (MH706706) as the reference. The x‑axis indicates the midpoint of each window, and the y‑axis shows nucleotide diversity (π). Functional regions are color‑coded as protein‑coding, rRNA‑coding, tRNA‑coding, or conserved non‑coding sequences.

**Table S1.** List of complete chloroplast genomes analyzed in this study.

| **Family** | **Genus** | **Species** | **GenBank accession number** | | **Length** |
| --- | --- | --- | --- | --- | --- |
| Pinaceae | *Abies* | *Abies balsamea* (L.) Mill. | | MH 706725 | 121,574 bp |
| Pinaceae | *Abies* | *Abies beshanzuensis* M. H. Wu | | MH 476330 | 121,399 bp |
| Pinaceae | *Abies* | *Abies beshanzuensis* var. *ziyuanensis* (L. K. Fu & S. L. Mo) L. K. Fu & Nan Li | | MH 706705 | 121,274 bp |
| Pinaceae | *Abies* | *Abies chensiensis* Tiegh. | | MH 706706 | 121,795 bp |
| Pinaceae | *Abies* | *Abies chensiensis* Tiegh. | | MH 047653 | 121,498 bp |
| Pinaceae | *Abies* | *Abies delavayi* Franch. | | MH 706709 | 120,141 bp |
| Pinaceae | *Abies* | *Abies delavayi* Franch. | | MK607413 | 120,142 bp |
| Pinaceae | *Abies* | *Abies delavayi* subsp. *fansipanensis*  (Q.P.Xiang, L.K.Fu & Nan Li) Rushforth | | MH 706720 | 120,093 bp |
| Pinaceae | *Abies* | *Abies delavayi* subsp. *fansipanensis*  (Q.P.Xiang, L.K.Fu & Nan Li) Rushforth | | MK607416 | 120,094 bp |
| Pinaceae | *Abies* | *Abies ernestii* Rehd. | | MH 706707 | 121841 bp |
| Pinaceae | *Abies* | *Abies ernestii* var. *salouenensis* (Bordères & Gaussen) W. C. Cheng & L. K. Fu | | MH 706708 | 121,759 bp |
| Pinaceae | *Abies* | *Abies fabri* (Mast.) Craib | | MH 706710 | 120,027 bp |
| Pinaceae | *Abies* | *Abies fanjingshanensis* W. L. Huang, Y. L. Tu & S. Z. Fang | | MH 706717 | 120,057 bp |
| Pinaceae | *Abies* | *Abies fargesii* Franch. | | MH706716 | 121,799 bp |
| Pinaceae | *Abies* | *Abies forrestii* C. C. Rogers | | MH 706715 | 120,022 bp |
| Pinaceae | *Abies* | *Abies georgei* var. *smithii* (Viguie et  Gaussen) Cheng et L | | NC_054152 | 121,191 bp |
| Pinaceae | *Abies* | *Abies kawakamii* (Hayata) T. Ito | | MH 706726 | 121,290 bp |
| Pinaceae | *Abies* | *Abies koreana* E. H. Wilson | | KP 742350 | 121,373 bp |
| Pinaceae | *Abies* | *Abies nephrolepis* (Trautv.) Maxim. | | KT 834974 | 121,336 bp |
| Pinaceae | *Abies* | *Abies nukiangensis* Cheng et L. K. Fu | | MH 706711 | 120,017 bp |
| Pinaceae | *Abies* | *Abies nukiangensis* Cheng et L. K. Fu | | MK607415 | 120,015 bp |
| Pinaceae | *Abies* | *Abies recurvata* Mast. | | MH706712* | 120,063 bp |
| Pinaceae | *Abies* | *Abies yuanbaoshanensis* Y. J. Lu & L. K. Fu | | MH 706718 | 121,795 bp |
| Pinaceae | *Keteleeria* | *Keteleeria davidiana* (Bertr.) Beissn. | | NC_011930^#^ | 117720 bp |

*: indicated newly generated chloroplast genomes; #：indicated outgroup.

**Table S2.** Gene organization of the *Abies recurvata* chloroplast genome.

| **Gene** | **Position** | **Length (bp)** | **Gene** | **Position** | **Length (bp)** | **Gene** | **Position** | **Length (bp)** |
| --- | --- | --- | --- | --- | --- | --- | --- | --- |
| *psbA* |  |  | *psbM* |  |  | *rps4* |  |  |
| *trnK-UUU* |  |  | *petN* |  |  | *trnS-GGA* |  |  |
| *matK* |  |  | *trnC-GCA* |  |  | *ycf3* |  |  |
| *chlB* |  |  | *rpoB* |  |  | *psaA* |  |  |
| *trnQ-UUG* |  |  | *rpoC1* |  |  | *psaB* |  |  |
| *psbK* |  |  | *rpoC2* |  |  | *rps14* |  |  |
| *psbI* |  |  | *rps2* |  |  | *trnfM-CAU* |  |  |
| *trnS-GCU* |  |  | *atpI* |  |  | *trnG-UCC* |  |  |
| *psaM* |  |  | *atpH* |  |  | *psbZ* |  |  |
| *ycf12* |  |  | *atpF* |  |  | *trnS-UGA* |  |  |
| *clpP* |  |  | *atpA* |  |  | *psbC* |  |  |
| *rps12* |  |  | *trnR-UCU* |  |  | *psbD* |  |  |
| *rpl20* |  |  | *trnG-GCC* |  |  | *trnT-GGU* |  |  |
| *rps18* |  |  | *ycf12* |  |  | *trnT-GGU* |  |  |
| *rpl33* |  |  | *psaM* |  |  | *rrn16* |  |  |
| *psaJ* |  |  | *trnS-GCU* |  |  | *trnI-GAU* |  |  |
| *trnP-UGG* |  |  | *psbB* |  |  | *trnA-UGC* |  |  |
| *trnW-CCA* |  |  | *psbT* |  |  | *rrn23* |  |  |
| *petG* |  |  | *psbN* |  |  | *rrn4.5* |  |  |
| *petL* |  |  | *psbH* |  |  | *rrn5* |  |  |
| *psbE* |  |  | *petB* |  |  | *trnR-ACG* |  |  |
| *psbF* |  |  | *petD* |  |  | *trnN-GUU* |  |  |
| *psbL* |  |  | *rpoA* |  |  | *chlL* |  |  |
| *psbJ* |  |  | *rps11* |  |  | *chlN* |  |  |
| *petA* |  |  | *rpl36* |  |  | *ycf1* |  |  |
| *cemA* |  |  | *infA* |  |  | *rps15* |  |  |
| *ycf4* |  |  | *rps8* |  |  | *psaC* |  |  |
| *psaI* |  |  | *rpl14* |  |  | *ccsA* |  |  |
| *accD* |  |  | *rpl16* |  |  | *trnL-UAG* |  |  |
| *trnR-CCG* |  |  | *rps3* |  |  | *trnP-GGG* |  |  |
| *rbcL* |  |  | *rpl22* |  |  | *rpl32* |  |  |
| *atpB* |  |  | *rps19* |  |  | *trnV-GAC* |  |  |
| *atpE* |  |  | *rpl2* |  |  | *rps7* |  |  |
| *trnM-CAU* |  |  | *rpl23* |  |  | *trnL-CAA* |  |  |
| *trnV-UAC* |  |  | *trnI-CAU* |  |  | *ycf2* |  |  |
| *trnE-UUC* |  |  | *trnF-GAA* |  |  | *trnH-GUG* |  |  |
| *trnY-GUA* |  |  | *trnL-UAA* |  |  | *trnI-CAU* |  |  |
| *trnD-GUC* |  |  | *trnT-UGU* |  |  |  |  |  |

**
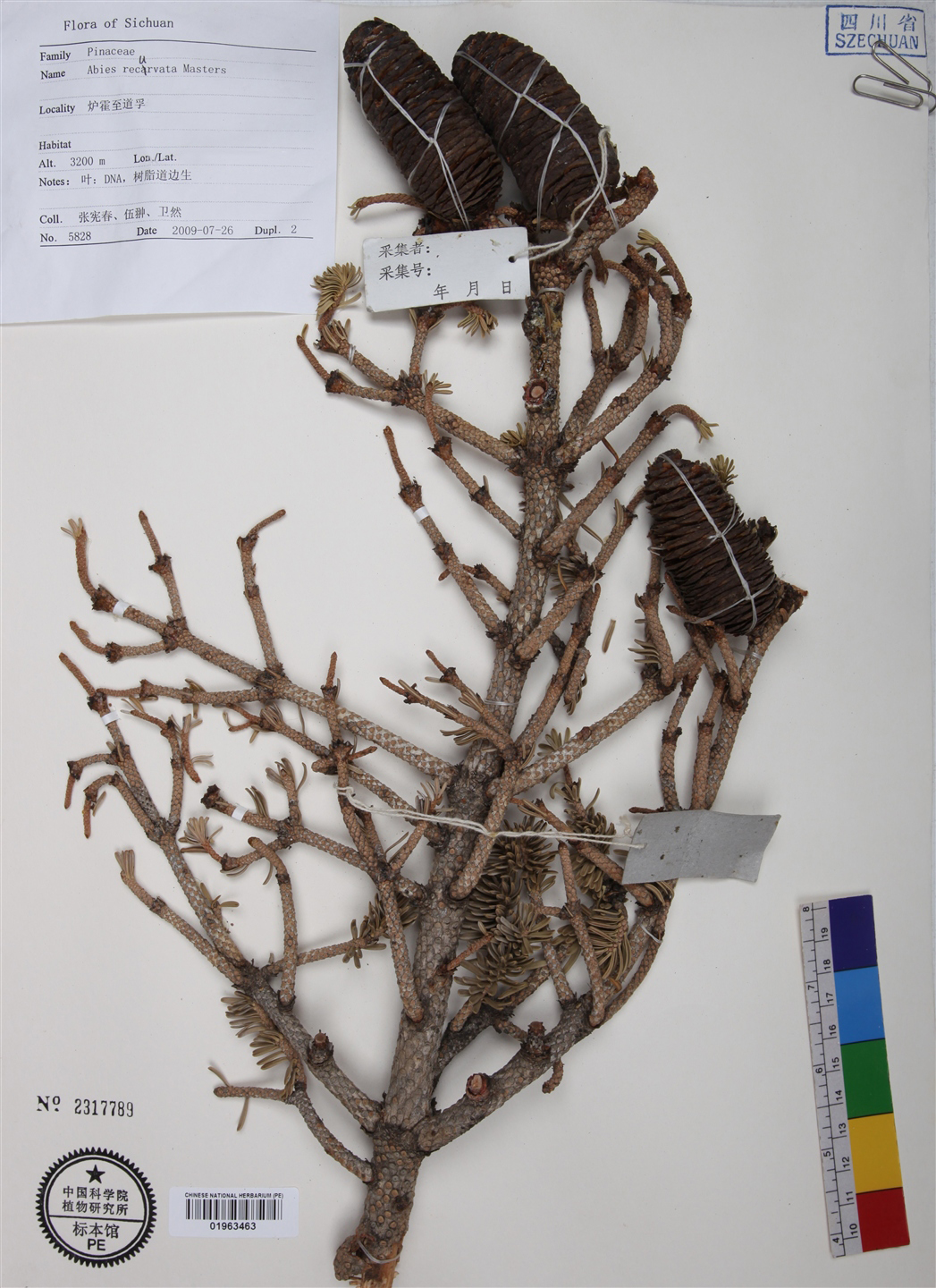
**

**Figure S1.** The illustration of *Abies recurvata* specimen preserved at the herbarium of the Institute of Botany, Chinese Academy of Sciences (PE). Its key taxonomic characteristics are as follows: bark irregularly plated; young shoots yellowish-tomentose; cones purplish, ellipsoid- to cylindrical-ovoid, with median seed scales reniform-flabellate and bract scales exserted.


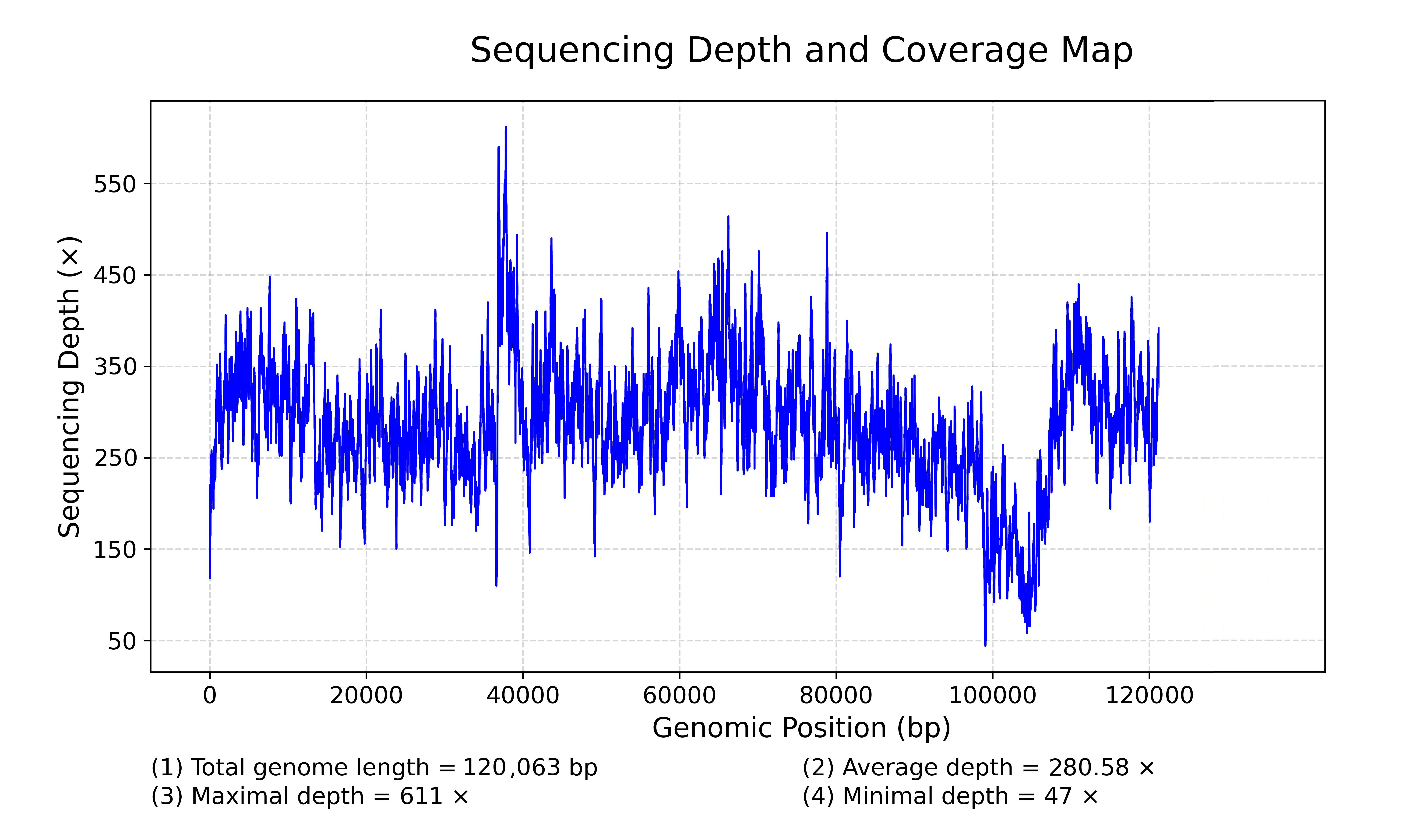


**Figure S2**. Coverage depth of the *Abies recurvata* chloroplast genome assembly, with an average of 280.58×, a maximum of 611×, and a minimum of 47×.


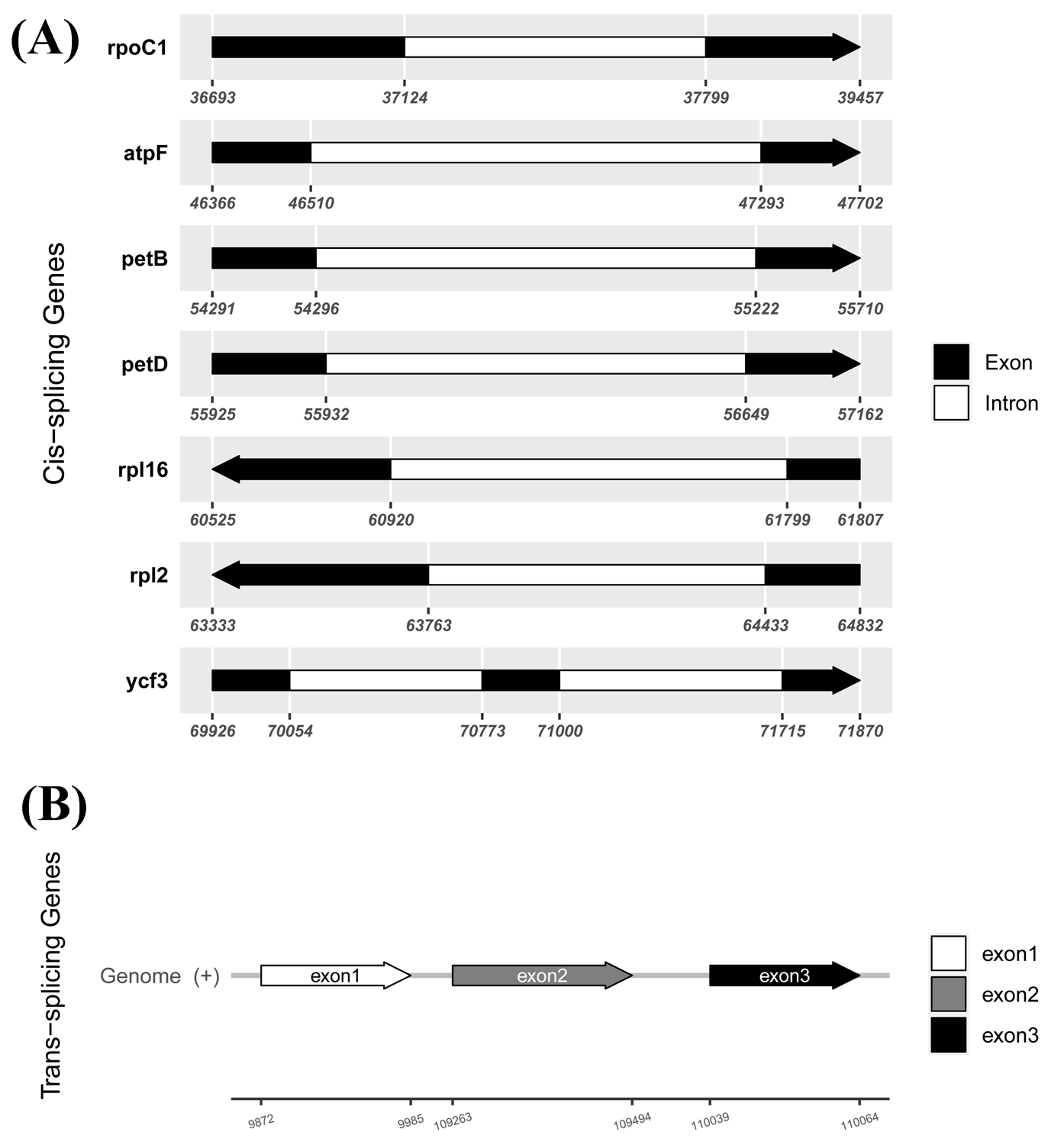


**Figure S3**. Schematic representation of cis- (A) and trans-splicing (B) genes in the chloroplast genome of *A. recurvata*.

**
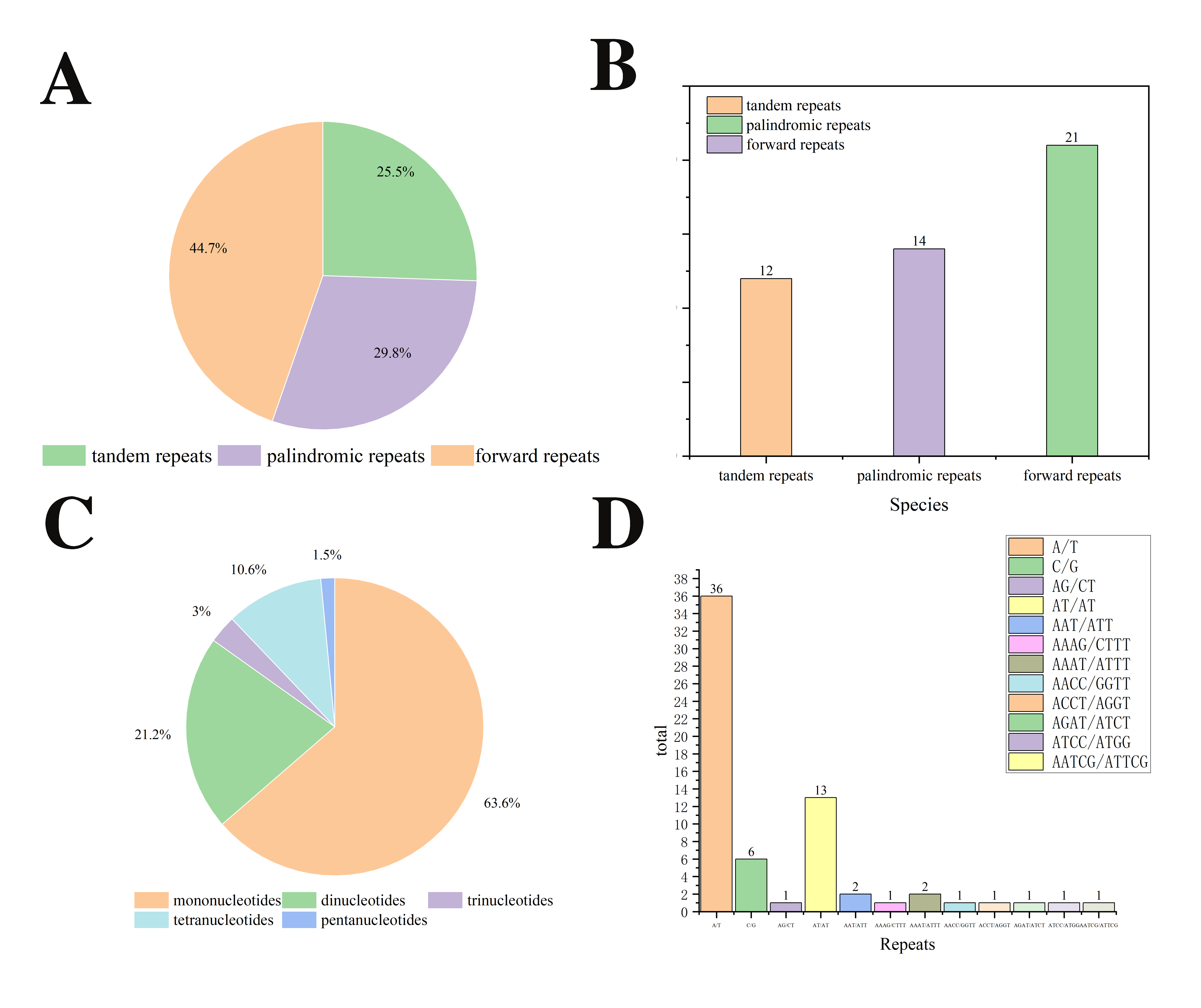
**

**Figure S4**. Types and amount of SSRs (A and B) and long sequence repeats (C and D) in the *Abies recurvata* chloroplast genome.

**
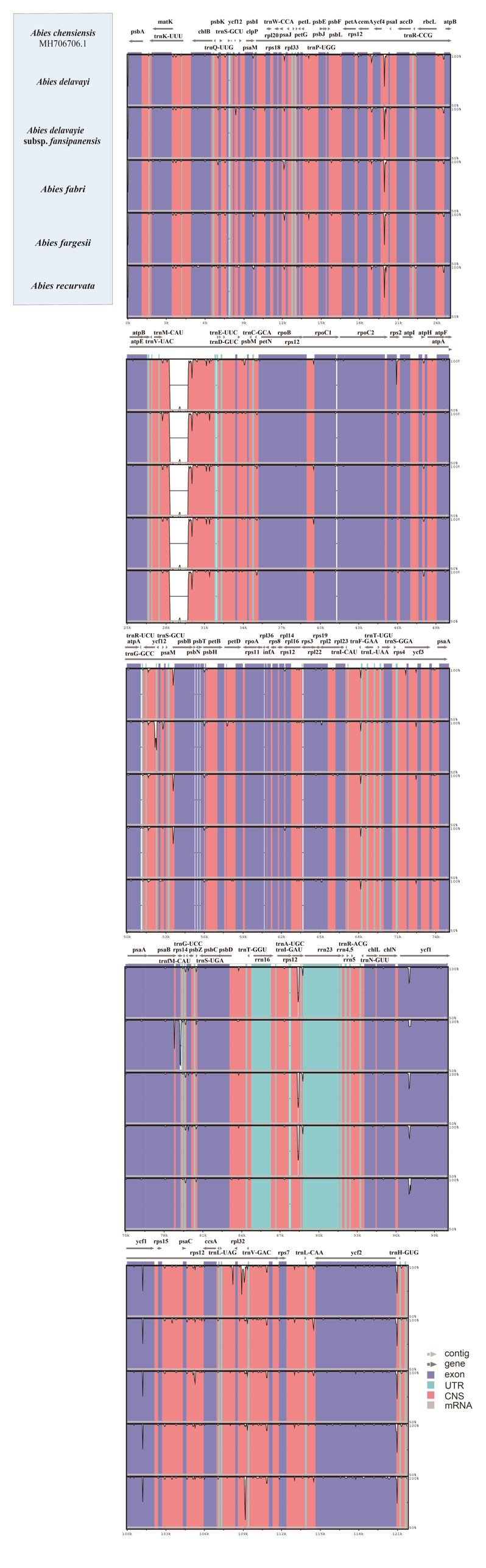
**

**
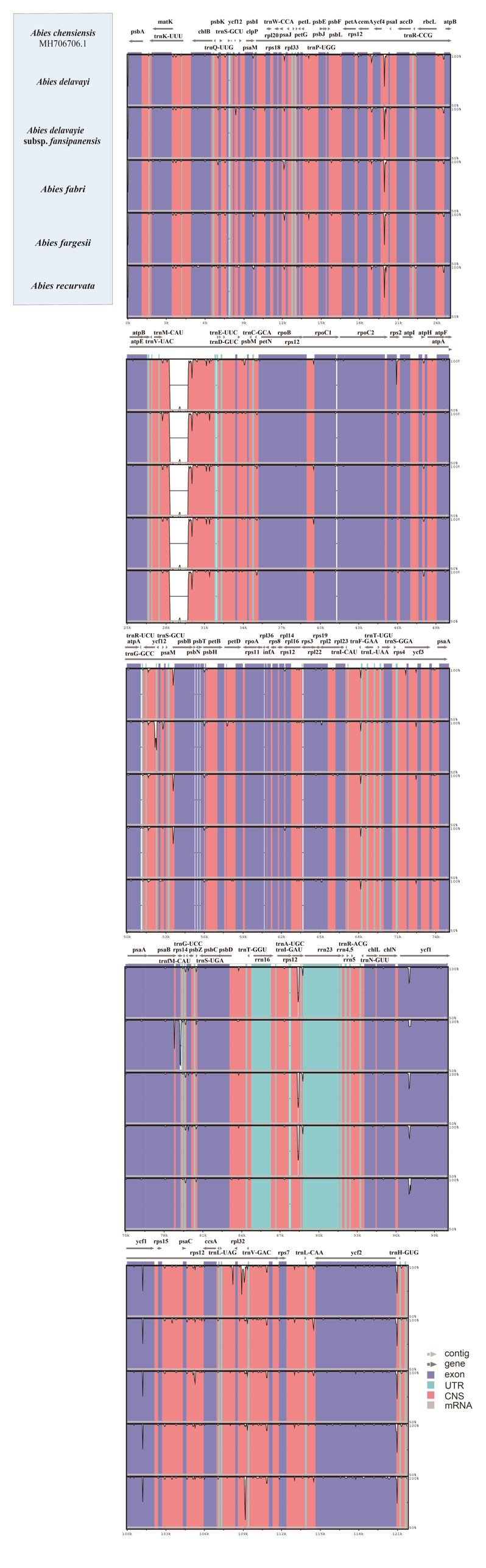
**

**
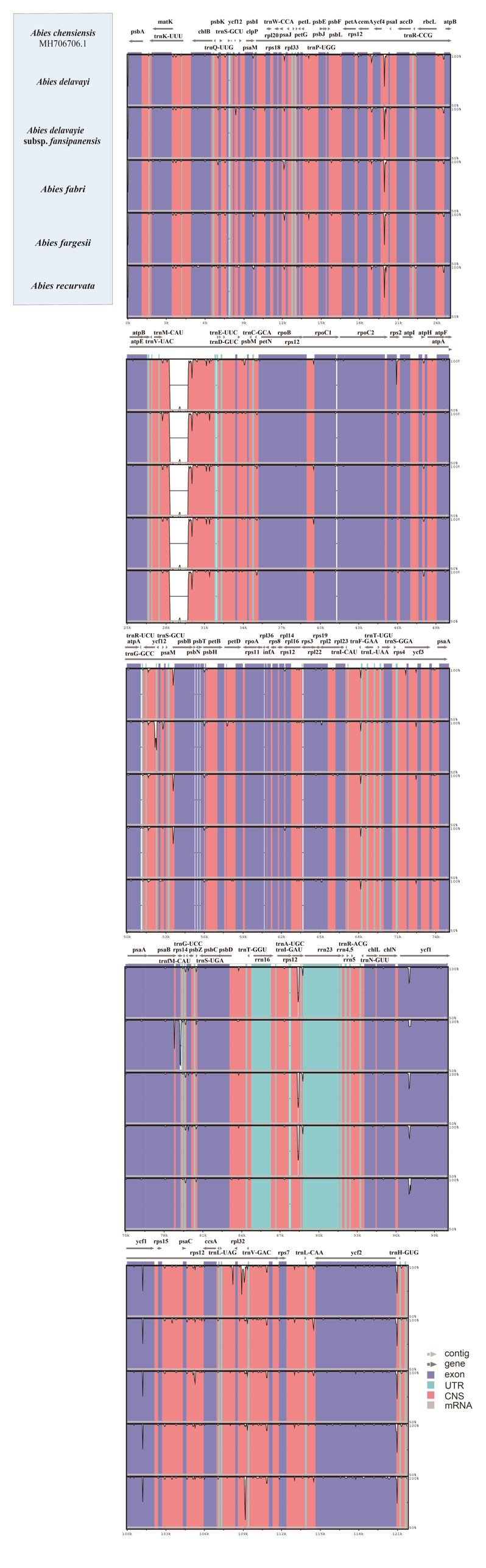
**

**Figure S5**. Comparison of six chloroplast genomes using the mVista alignment program, with *Abies chensiensis* (MH706706) as a reference. The X-axis means the window's midpoint, and the y-axis means nucleotide diversity (Pi). Genome regions are colour-coded as protein-coding, rRNA coding, tRNA coding, or conserved noncoding sequences.
